# A Correction

**Published:** 1891-12

**Authors:** Fred. A. Levy

**Affiliations:** Secretary N.A.D.E.


### A CORRECTION.

To the Editor:

My attention has been called to the fact that the name of the dental department of the National University of Washington, D. C., does not appear in the list of colleges which the National Association of Dental Examiners recommends to the various State Boards to accept as reputable. In justice to that college I would state that the omission is an error, the correction of which the State Board will please take notice.

Fred. A. Levy,
*Secretary N.A.D.E.*

November 12, 1891.

